# Association of raisin consumption with nutrient intake, diet quality, and health risk factors in US adults: National Health and Nutrition Examination Survey 2001–2012

**DOI:** 10.1080/16546628.2017.1378567

**Published:** 2017-09-24

**Authors:** Victor L. Fulgoni, James Painter, Arianna Carughi

**Affiliations:** ^a^ Nutrition Impact, LLC, Battle Creek, MI, USA; ^b^ School of Public Health, University of Texas, Brownsville, TX, USA; ^c^ Sun-Maid Growers of California, Kingsburg, CA, USA

**Keywords:** NHANES, raisins, Healthy Eating Index, body weight, BMI, obesity, metabolic syndrome

## Abstract

Raisins are one of the most commonly consumed dried fruits. Because of their unique nutrient profile, raisins may have some distinctive health benefits. The purpose of this study was to examine the cross-sectional association between raisin consumption and nutrient intake, dietary quality, body weight, and metabolic syndrome risk factors in adults. Data from the National Health and Nutrition Examination Survey (NHANES), 2001–2012 (*n* = 29,684) were used. Raisin consumers (*n* = 458, 60% female) were defined as those having any amount of raisins during the first 24 h dietary recall. Diet quality was calculated using the Healthy Eating Index-2010 (HEI-2010). Covariate (demographic and lifestyle)-adjusted regression analyses were conducted using appropriate sample weights and significance was set at *p* < 0.01. Raisin consumers had higher intakes of energy (9%); higher intakes of ‘nutrients of public health concern/shortfall nutrients’, such as dietary fiber (34%), potassium (16%), magnesium (22%), vitamin C (24%), and vitamin E (22%); and lower intakes of ‘nutrients to limit’, such as added sugar (−17%), saturated fat (−15%), and sodium (−10%), than non-consumers. No associations were observed for intakes of calcium, iron, vitamin A, vitamin D, and folate. Consumers had higher intakes of total fruit (72%), whole fruit (111%), vegetables (22%), and whole grains (109%), and had a higher diet quality, as indicated by 25% higher total HEI-2010 scores than non-consumers. Compared to non-consumers, raisin consumers had a lower body weight (−4.2%), body mass index (−5.2%), and waist circumference (−3.8%), were 39% less likely to be overweight or obese, and had a 54% reduced risk of metabolic syndrome. In conclusion, raisin consumption was associated with better nutrient intake, diet quality, and weight parameters, and with lower risk of being obese and having metabolic syndrome in US adults.

## Introduction

Dietary guidelines around the world unequivocally recommend increased consumption of fruit and vegetables. The Dietary Guidelines for Americans (DGA) 2015–2020 also recommend the consumption of fruit and vegetables as part of a healthy eating pattern [1]. MyPlate (ChooseMyPlate.gov) indicates that half of the food on a meal plate should be fruit and vegetables and recommends 1.5–2 cups equivalent of fruit per day for adults, depending on age, gender, and physical activity [2]. Fruits are an important source of many essential nutrients, including potassium, dietary fiber, vitamin C, and folate, and are naturally low in fat, sodium, and calories. Despite these recommendations, over 75% of adult females and 85% of adult males in the USA do not consume an adequate amount of fruit and only 12% of adult females and 6% of adult males consume the recommended daily servings of fruit [3]. The DGA identified vitamin A, vitamin D, vitamin E, folate, vitamin C, calcium, iron (for certain age and gender groups), magnesium, potassium, and fiber as ‘shortfall nutrients’, since these nutrients are being consumed at levels below those recommended; and of these, vitamin D, calcium, iron, potassium, and fiber are being underconsumed to the extent that may pose a public health concern and are thus described as ‘nutrients of public health concern’ [1].

Raisins are dried grapes and are one of the most commonly consumed dried fruits [4]. Raisins are consumed across the globe, and because of their unique nutrient profile they may have some distinctive health benefits. Raisins are low–medium energy dense, and provide important minerals and dietary fiber including fructooligosaccharides. One snack serving (43 g) of raisins contributes 129 kcal, 1.6 g dietary fiber, 0.2 g total fat, 25 g total sugar, 322 mg potassium, 14 mg magnesium, and 0.8 mg iron [5]. Raisins also provide a wide variety of phytochemicals including flavonoids (catechins, kaempferol, quercetin, and rutin), hydrocinnamic acids (cafteric and coutaric acids), epicatechins, phytoestrogens (daidzein and genestein), and resveratrol [6,7]. The effects of raisin intake on blood pressure, lipid profile, glucose, postprandial glycemia/insulinemia, oxidative damage, and satiety have been previously investigated in human intervention studies [7–15]. Epidemiological studies examining the effect of raisins on diet quality and markers of health are very limited. Intake of dried fruits including raisins and other dried fruits, as well as intake of grape products (grapes, grape, juice and raisins), was associated with a higher intake of key nutrients and healthier dietary patterns in two previous analyses of data from the National Health and Nutrition Examination Survey (NHANES), but neither specifically focused on raisins (although raisins were a large component of dried fruit in one study) [4,16]. Given that raisins were the predominant dried fruit consumed in the studies mentioned above, we wanted to assess whether the results were due specifically to raisin consumption. Thus, the purpose of this study was to examine the cross-sectional association between raisin consumption and nutrient intakes, diet quality, body weight, and metabolic syndrome risk factors in a nationally representative large sample of US adults.

## Methods

### Subjects

Data from What We Eat In America 2001–2012, the dietary intake component of the NHANES, were used to assess raisin intake [17]. NHANES is a continuous survey conducted by the National Center for Health Statistics (NCHS). The present analysis combined six NHANES data sets (NHANES 2001–2002, 2003–2004, 2005–2006, 2007–2008, 2009–2010, and 2011–2012). Adults aged 19 years and older (*n* = 29,684) with reliable 24 h dietary recall interviews were included. Pregnant and/or lactating females and those with incomplete or unreliable 24 h recall data were excluded. All participants provided written informed consent and the Research Ethics Review Board at the NCHS approved the survey protocol. Dietary intake data were obtained using the United States Department of Agriculture’s (USDA’s) automated multiple-pass method [17] in the Mobile Examination Center. Data were collected to represent all days of the week and food models were used to help with portion size determination. For these analyses, only subjects with reliable 24 h dietary recall interviews (day 1 data only) as determined by USDA staff were used.

### Estimation of intake

Raisin intakes were assessed using two USDA food codes for raisins [18]: plain raisins (USDA food code 62125100) and cooked raisins (USDA food code 62125110). Raisin consumers (*n* = 458) were defined as those consuming any amount of raisins during the first 24 h recall. Energy and nutrient intake were determined using the USDA Nutrient Database for Standard Reference Releases [19] in conjunction with the respective Food and Nutrient Database for Dietary Studies for participants in each NHANES cycle [18]. The USDA MyPyramid Equivalents Database (MPED) and Food Patterns Equivalents Database (FPED) were used to calculate intake as MyPlate [20,21] servings. The MPED and FPED translate dietary recall data into equivalent servings of the MyPlate major food groups and corresponding subgroups. The number of MyPlate servings was aggregated over all foods consumed during the 24 h recall to calculate the MyPlate food group intakes per day.

### Estimation of diet quality

Diet quality was calculated using the Healthy Eating Index-2010 (HEI-2010). HEI-2010 has 12 components, each representing a different aspect of diet quality [22]. Nine component scores, namely total fruit, whole fruit, total vegetables, greens and beans, whole grains, dairy, total protein foods, seafood and plant proteins, and fatty acid ratio, measure adequacy and a higher score indicates higher consumption; three component scores, namely refined grains, sodium, and empty calories, measure moderation items and higher scores indicate lower consumption. Scores on many of the HEI components are based on intake per 1000 kcal, so higher scores cannot be achieved just by eating more of everything. HEI-2010 scores were estimated using day 1 dietary intake data. The SAS code used to calculate HEI-2010 scores was downloaded from the USDA website [23].

### Estimation of physiological markers of risk

Health indices evaluated included body weight, body mass index (BMI), waist circumference, skinfold thickness, blood pressure (BP), fasting plasma glucose, fasting plasma insulin, homeostatic model assessment of insulin resistance (HOMA-IR), C-reactive protein (CRP), fasting triglycerides, total cholesterol, low-density lipoprotein (LDL)-cholesterol (fasting), high-density lipoprotein (HDL)-cholesterol, and homocysteine, using NHANES standard protocols [17]. For a variety of reasons, not all individuals have values for all tests (see tables for sample numbers). Risk factors for physiological variables were defined as obesity (BMI ≥ 30 kg/m^2^), overweight (BMI 25–29.9 kg/m^2^), elevated waist circumference (> 102 cm for males and > 88 cm for females); elevated BP (systolic BP ≥ 130 mmHg or diastolic BP ≥ 85 mmHg); elevated total cholesterol (≥ 200 mg/dL), reduced HDL-cholesterol (< 40 mg/dL for males and < 50 mg/dL for females); elevated LDL-cholesterol (≥ 100 mg/dL), elevated triglycerides (≥ 150 mg/dL); elevated CRP (≥ 6.0 mg/dL); elevated glucose (fasting glucose ≥ 110 mg/dL); elevated insulin; elevated HOMA-IR (≥ 4.0); and metabolic syndrome (NHLBI Adult Treatment Panel III criteria, namely having three or more of the following risk factors: elevated waist circumference, elevated BP or taking medication, reduced HDL-cholesterol, elevated triglycerides, and elevated glucose or taking medication) [24–26].

### Statistical analysis

Least square means (LSMs) and standard errors (SEs) were determined for energy and nutrient intakes, food group intake, diet quality, and physiological markers of metabolic disease risk in raisin consumers and non-consumers via regression analyses. The data were adjusted for the complex sample design of NHANES using appropriate survey weights, strata, and primary sampling units. To account for NHANES national probability sampling and to ensure that estimates remain nationally representative, sample weights must be used in all analyses: day 1 dietary weights were used in all intake analyses, while the Mobile Examination Center weights were used for physiological variables except where the outcome was a fasting laboratory variable, in which case fasting subsample weights were used. Food group/nutrient intakes were adjusted for age, gender, ethnicity, poverty–income ratio, physical activity level, current smoking status, alcohol intake, and energy intake (except for energy intake). Diet quality was adjusted for the same covariates but without energy intake, as HEI scores are already adjusted for energy intake. Physiological variables were adjusted for age, gender, ethnicity, poverty–income ratio, self-reported physical activity level (categorized as sedentary, moderate, or vigorous based on responses to questions on activity), current smoking status, alcohol intake, and BMI (for non-weight-related variables). Logistic regression was used to assess the association of raisin consumption with elevated/reduced risk of various health-related parameters; covariates for these analyses were similar to those for physiological variables. SAS 9.2 (SAS Institute, Cary, NC, USA) and SUDAAN 11 (RTI, Research Triangle Park, NC, USA) were used for all analyses. Data are presented as LSM ± SE and significance was set at *p* < 0.01.

## Results

Approximately 1.76% of adults (2.08% of females and 1.41% of males) aged 19 years and older (*n* = 458, 60% female) were raisin consumers. The amount of raisins consumed was 32.5 ± 1.9 g/day for both genders combined and 26.3 ± 2.2 g/day and 41.8 ± 3.6 g/day for females and males, respectively. Raisin consumers were more likely to be older and non-Hispanic white, and to have a higher income; and less likely to be male, Mexican American, and smokers, and to have a sedentary lifestyle compared to non-consumers (Table 1).

**Table 1.** Characteristics of adult raisin consumers, National Health and Nutrition Examination Survey (NHANES) 2001–2012.

Variables	Non-consumers	Consumers	*p*^a^
Sample (*n*)	29,226	458	
Age (years)	46.4 ± 0.3	54.1 ± 1.2	< 0.0001
Gender			
Male (%)	49.4 ± 0.3	39.9 ± 3.2	0.0033
Race/ethnicity			
Mexican American (%)	7.94 ± 0.66	3.78 ± 0.99	0.0005
Other Hispanic (%)	4.66 ± 0.50	3.69 ± 0.67	0.2459
Non-Hispanic white (%)	70.3 ± 1.4	81.5 ± 2.1	< 0.0001
Non-Hispanic black (%)	11.4 ± 0.8	7.87 ± 1.28	0.0165
Other (%)	5.66 ± 0.32	3.14 ± 1.02	0.0186
Poverty–income ratio	2.97 ± 0.03	3.44 ± 0.12	0.0003
Smoker (%)	24.0 ± 0.5	8.18 ± 1.88	< 0.0001
Physical activity			
Sedentary (%)	27.7 ± 0.6	19.6 ± 2.1	0.0002
Moderate (%)	34.9 ± 0.4	39.5 ± 4.1	0.2657
Vigorous (%)	37.4 ± 0.7	40.9 ± 3.8	0.3584

Data are shown as mean ± SE.

^a^ *p* value for difference between raisin consumers and non-consumers.

There were significant differences in nutrient intakes between the raisin consumers and the non-consumers (Table 2). Compared to non-consumers, raisin consumers had significantly higher (*p* < 0.01) intakes of calories (8.8%), and energy-adjusted daily intakes of carbohydrate (7.8%), dietary fiber (34.0%), total sugar (11.3%), copper (20.3%), magnesium (22.4%), potassium (15.7%), β-carotene (51.2%), vitamin C (23.6%), vitamin E (22.0%), and vitamin K (52.9%). Adult raisin consumers also had significantly lower (*p* < 0.01) intakes of added sugar (−17.1%), total fat (−7.6%), monounsaturated fatty acids (MUFA) (−8.7%), saturated fatty acids (SFA) (−14.8%), cholesterol (−22.4%), and sodium (−9.9%) compared to non-consumers. The intake of other nutrients was not significantly different between raisin consumers and non-consumers (Table 2).

**Table 2.** Energy and nutrient intakes in adult raisin consumers (*n* = 458) and non-consumers (*n* = 29,226): National Health and Nutrition Examination Survey (NHANES) 2001–2012, gender-combined data.

Variables^a^	Non-consumers	Consumers	*p*^b^
Energy (kcal)	2074 ± 9	2257 ± 44	0.0001
Protein (g)	82.2 ± 0.3	81.0 ± 1.5	0.4176
Carbohydrate (g)	261 ± 1	282 ± 4	< 0.0001
Dietary fiber (g)	16.5 ± 0.1	22.1 ± 0.8	< 0.0001
Total sugars (g)	116 ± 1	129 ± 4	0.0011
Added sugars (tsp)	18.0 ± 0.2	14.9 ± 0.7	0.0001
Total fat (g)	78.0 ± 0.3	72.1 ± 1.6	0.0004
MUFA (g)	28.8 ± 0.1	26.3 ± 0.7	0.0005
PUFA (g)	17.3 ± 0.1	18.1 ± 0.6	0.1921
SFA (g)	25.0 ± 0.1	21.3 ± 0.9	< 0.0001
Cholesterol (mg)	298 ± 2	231 ± 11	< 0.0001
Calcium (mg)	861 ± 6	886 ± 28	0.4032
Copper (mg)	1.33 ± 0.02	1.60 ± 0.05	< 0.0001
Iron (mg)	15.1 ± 0.2	16.2 ± 0.4	0.0106
Magnesium (mg)	290 ± 1	355 ± 11	< 0.0001
Phosphorus (mg)	1312 ± 5	1364 ± 27	0.0657
Potassium (mg)	2665 ± 10	3084 ± 77	< 0.0001
Selenium (µg)	112 ± 1	106 ± 3	0.0363
Sodium (mg)	3541 ± 17	3190 ± 78	< 0.0001
Zinc (mg)	11.7 ± 0.2	11.6 ± 0.4	0.9154
Vitamin A (µg)	589 ± 12	653 ± 36	0.0939
β-Carotene (µg)	2119 ± 50	3204 ± 340	0.0025
Thiamin (mg)	1.59 ± 0.02	1.62 ± 0.05	0.5114
Riboflavin (mg)	2.03 ± 0.03	2.04 ± 0.07	0.8993
Niacin (mg)	24.4 ± 0.1	24.4 ± 0.6	0.9967
Total folate (µg)	405 ± 3	432 ± 14	0.0648
Vitamin B_6_ (mg)	1.98 ± 0.03	2.12 ± 0.08	0.0972
Vitamin B_12_ (µg)	5.21 ± 0.18	4.48 ± 0.30	0.0114
Vitamin C (mg)	94.4 ± 1.2	117 ± 8	0.0081
Vitamin D (µg)	4.45 ± 0.15	4.06 ± 0.34	0.2297
Vitamin E (mg)	7.31 ± 0.12	8.92 ± 0.47	0.0007
Vitamin K (µg)	102 ± 2	156 ± 19	0.0073

Data are shown as least square mean ± SE.

^a^Values were adjusted for age, gender, ethnicity, poverty–income ratio, physical activity level, current smoking status, alcohol, and energy intake (except for energy).

^b^ *p* value for difference between raisin consumers and non-consumers.

MUFA, monounsaturated fatty acids; PUFA, polyunsaturated fatty acids; SFA, saturated fatty acids.

Intake of raisins was also associated with significant differences (*p* < 0.01) in specific MyPlate food groups (Table 3). Significantly higher intakes of total fruit (72.1%), whole fruit (112%), total vegetable (21.9%), and whole grain (109%) were observed among adults consuming raisins compared to non-consumers. Raisin intake was also associated with a significantly better overall diet quality among consumers, as indicated by their 25.1% higher HEI-2010 total score (61.4 ± 1.0 vs 49.1 ± 0.2 points, *p* < 0.0001) compared to those not consuming raisins (Table 4). Raisin consumers also had higher (*p* < 0.01) HEI-2010 component scores for total vegetables (11.8%), greens and beans (40.9%), total fruit (56.7%), whole fruit (83.0%), whole grain (102%), seafood and protein (44.4%), sodium (30.8%), refined grains (16.0%), and calories from solid fats, alcohol, and added sugars (SoFAAS) (18.7%) compared to non-consumers (Table 4).

**Table 3.** Intake of MyPlate food groups in adult raisin consumers (*n* = 458) and non-consumers (*n* = 29,226): National Health and Nutrition Examination Survey (NHANES) 2001–2012, gender-combined data.

Variables^a^	Non-consumers	Consumers	*p*^b^
Total fruit (cup eq.)	1.07 ± 0.04	1.84 ± 0.11	< 0.0001
Whole fruit (cup eq.)	0.66 ± 0.03	1.39 ± 0.10	< 0.0001
Total vegetable (cup eq.)	1.54 ± 0.03	1.88 ± 0.10	0.0005
Total grain (oz eq.)	6.73 ± 0.09	6.66 ± 0.21	0.7669
Whole grain (oz eq.)	0.70 ± 0.04	1.47 ± 0.10	< 0.0001
Total dairy (cup eq.)	1.36 ± 0.05	1.38 ± 0.09	0.8195
Total protein group (oz eq.)	6.27 ± 0.11	6.14 ± 0.24	0.5817

Data are shown as least square mean ± SE.

^a^Values were adjusted for age, gender, ethnicity, poverty–income ratio, physical activity level, current smoking status, alcohol, and energy (kcal).

^b^ *p* value for difference between raisin consumers and non-consumers.

**Table 4.** Healthy Eating Index-2010 (HEI-2010) total score and component scores of adult raisin consumers (*n* = 458) and non-consumers (*n* = 29,226): National Health and Nutrition Examination Survey (NHANES) 2001–2012, gender-combined data.

Variables^a^	Non-consumers	Consumers	*p*^b^
HEI-2010 total score	49.07 ± 0.20	61.40 ± 0.97	< 0.0001
Component 1 (total vegetables)	3.09 ± 0.02	3.45 ± 0.11	0.0017
Component 2 (greens & beans)	1.37 ± 0.03	1.93 ± 0.17	0.0013
Component 3 (total fruit)	2.31 ± 0.03	3.62 ± 0.11	< 0.0001
Component 4 (whole fruit)	2.09 ± 0.03	3.83 ± 0.09	< 0.0001
Component 5 (whole grains)	2.08 ± 0.04	4.21 ± 0.20	< 0.0001
Component 6 (dairy)	4.41 ± 0.04	4.58 ± 0.25	0.5022
Component 7 (total protein foods)	4.31 ± 0.02	4.22 ± 0.08	0.3107
Component 8 (seafood & plant protein)	1.99 ± 0.03	2.87 ± 0.17	< 0.0001
Component 9 (fatty acid ratio)	5.39 ± 0.05	6.17 ± 0.31	0.0112
Component 10 (sodium)	4.25 ± 0.05	5.56 ± 0.27	< 0.0001
Component 11 (refined grains)	5.72 ± 0.04	6.64 ± 0.25	0.0004
Component 12 (SoFAAS calories)	12.06 ± 0.11	14.31 ± 0.38	< 0.0001

Data are shown as least square mean ± SE.

^a^Values were adjusted for age, gender, ethnicity, poverty–income ratio, physical activity level, current smoking status, and alcohol.

^b^ *p* value for difference between raisin consumers and non-consumers.

SoFAAS, solid fats, alcohol, and added sugars.

Adult raisin consumers had slightly but significantly lower body weight (−4.2%, *p* = 0.0056), BMI (−5.2%, *p* = 0.0004), and waist circumference (−3.8%, *p* = 0.0003) compared to non-consumers (Table 5). There were no significant differences in physiological laboratory measures (systolic BP, diastolic BP, total cholesterol, LDL-cholesterol, HDL-cholesterol, triglycerides, CRP, plasma glucose, insulin, or HOMA-IR) associated with raisin consumption in adults (Table 5). The differences between consumers and non-consumers remained non-significant for these physiological variables (*p* > 0.01) when the data were further analyzed separately for males and females (data not presented).

**Table 5.** Association of raisin consumption with anthropometric and physiological variables in adults: National Health and Nutrition Examination Survey (NHANES) 2001–2012, gender-combined data.

Variables^a^	*n*	Non-consumers	Consumers	*p*^b^
Anthropometric variables				
Body weight (kg)	29,255	79.8 ± 0.3	76.4 ± 1.2	0.0056
Body mass index (kg/m^2^)	29,115	28.8 ± 0.1	27.3 ± 0.4	0.0004
Waist circumference (cm)	28,519	97.8 ± 0.2	94.1 ± 1.0	0.0003
Triceps skinfold thickness (mm)	22,355	19.2 ± 0.1	17.8 ± 0.6	0.0157
Waist to height ratio	28,410	0.59 ± 0.004	0.56 ± 0.01	0.0001
Physiological laboratory variables				
Diastolic blood pressure (mmHg)	28,496	71.3 ± 0.2	71.4 ± 0.7	0.9949
Systolic blood pressure (mmHg)	28,615	125 ± 0.3	123 ± 1	0.1623
Total cholesterol (mg/dL)	28,076	199 ± 1	198 ± 3	0.9010
LDL-cholesterol (mg/dL)	13,306	117 ± 1	115 ± 3	0.3731
HDL-cholesterol (mg/dL)	28,075	52.3 ± 0.2	54.4 ± 1.7	0.2315
Triglyceride (mg/dL)	13,772	140 ± 2	131 ± 6	0.0951
CRP (mg/dL)	23,604	0.43 ± 0.01	0.37 ± 0.47	0.1694
Plasma glucose (mg/dL)	13,903	107 ± 1	104 ± 1.5	0.0191
Insulin (µU/mL)	13,639	12.6 ± 0.2	11.8 ± 0.5	0.0714
HOMA-IR	13,618	3.52 ± 0.06	3.20 ± 0.15	0.0335

Data are shown as least square mean ± SE.

^a^Values were adjusted for age, gender, ethnicity, poverty–income ratio, physical activity level, current smoking status, alcohol, and BMI (only for variables not related to weight).

^b^ *p* value for difference between raisin consumers and non-consumers.

LDL, low-density lipoprotein; HDL, high-density lipoprotein; CRP, C-reactive protein; HOMA-IR, homeostatic model assessment of insulin resistance.

Consumption of raisins was also associated with a 37% reduced risk of being overweight [odds ratio (OR) = 0.63, 99% confidence interval (CI) 0.42, 0.95; *p* = 0.0037], 43% reduced risk of being obese (OR = 0.57, 99% CI 0.35, 0.93; *p* = 0.0032), 39% reduced risk of being overweight or obese (OR = 0.61, 99% CI 0.41, 0.89; *p* = 0.0009), 48% reduced risk of having an elevated waist circumference (OR = 0.52, 99% CI 0.36, 0.74; *p* < 0.0001), 95% reduced risk of having elevated CRP (OR = 0.05, 99% CI 0.01, 0.34; *p* < 0.0001), and 54% reduced risk of metabolic syndrome (OR = 0.46, 99% CI 0.26, 0.82; *p* = 0.0005). The odds ratios of other health-related conditions were similar (*p* > 0.01) for raisin consumers compared to non-consumers (Table 6) and remained similar when data were further analyzed for males and females separately (data not presented).

**Table 6.** Association of raisin consumption with odds ratios of weight/waist status and other risk factors in adults: National Health and Nutrition Examination Survey (NHANES) 2001–2012, gender-combined data.

Variables^a,b^	*n*	Odds ratio (99% CI)		
		Non-consumers	Consumers	*p*^c^
Overweight	18,999	1.00	0.63 (0.42, 0.95)	0.0037
Obese	19,220	1.00	0.57 (0.35, 0.93)	0.0032
Overweight or obese	29,115	1.00	0.61 (0.41, 0.89)	0.0009
Elevated waist circumference	28,519	1.00	0.52 (0.36, 0.74)	< 0.0001
Elevated blood pressure	28,868	1.00	0.70 (0.49, 1.01)	0.0122
Elevated total cholesterol	28,337	1.00	0.75 (0.50, 1.12)	0.0614
Reduced HDL-cholesterol	28,336	1.00	0.77 (0.52, 1.13)	0.0745
Elevated LDL-cholesterol	16,057	1.00	0.96 (0.57, 1.62)	0.8342
Elevated triglycerides	16,432	1.00	0.76 (0.44, 1.32)	0.2038
Elevated CRP	23,604	1.00	0.05 (0.01, 0.34)	< 0.0001
Elevated glucose	15,426	1.00	0.59 (0.33, 1.07)	0.0220
Elevated insulin	15,204	1.00	0.66 (0.32, 1.35)	0.1320
Elevated HOMA-IR	15,188	1.00	0.59 (0.30, 1.18)	0.0517
Metabolic syndrome	20,759	1.00	0.46 (0.26, 0.82)	0.0005

^a^Risk factors for physiological variables were defined as obesity [body mass index (BMI) ≥ 30 kg/m^2^], overweight (BMI 25–29.9 kg/m^2^), elevated waist circumference (> 102 cm for males and > 88 cm for females); elevated blood pressure (systolic BP ≥ 130 mmHg or diastolic BP ≥ 85 mmHg); elevated total cholesterol (≥ 200 mg/dL), reduced high-density lipoprotein (HDL)-cholesterol (< 40 mg/dL for males and < 50 mg/dL for females); elevated low-density lipoprotein (LDL)-cholesterol (≥ 100 mg/dL), elevated triglycerides (≥ 150 mg/dL); elevated C-reactive protein (CRP) (≥ 6.0 mg/dL); elevated glucose (fasting glucose ≥ 110 mg/dL); elevated insulin; elevated homeostatic model assessment of insulin resistance (HOMA-IR) (≥ 4.0); and metabolic syndrome (National Heart, Lung, and Blood Institute Adult Treatment Panel III criteria, namely having three or more of the following risk factors: elevated waist circumference, elevated BP, reduced HDL-cholesterol, elevated triglycerides, elevated glucose) [24–26].

^b^Values were adjusted for age, gender, ethnicity, poverty–income ratio, physical activity level, current smoking status, alcohol, and BMI (only for variables not related to weight).

^c^ *p* value for difference between raisin consumers and non-consumers.

## Discussion

This is the first report to specifically investigate raisin (plain or cooked) consumption in the US population and to explore its cross-sectional relationships with nutrient intake, diet quality, and physiological markers of health using a large national representative sample of US adults. In the present study, we combined data from six recent NHANES cycles, and the combined data set provided a sample size of over 29,000 adults. The NHANES 2001–2012 data showed that 1.5% of the adult US population consumed raisins on the day of the recall and raisin consumption was associated with better nutrient intake, diet quality, and weight and metabolic syndrome parameters.

Adult consumers of raisins consumed significantly more calories and carbohydrate but less fat compared to their respective non-consumers. In addition, they consumed more energy-adjusted dietary fiber, copper, magnesium, potassium, β-carotene, vitamin C, vitamin E, and vitamin K compared to non-consumers. A recent analysis of NHANES 2003–2008 also reported higher intake of several key nutrients including dietary fiber, vitamin A, vitamin C, calcium, magnesium, and potassium among consumers of grape products (fruit, juice, and raisins) compared to non-consumers [16]. Higher intakes of most vitamins and minerals were also reported for consumers of dried fruits compared to non-consumers in an earlier analysis of NHANES 1994–2004 [4]. Most of these nutrients (fiber, magnesium, potassium, vitamin C, and vitamin E) are currently underconsumed and are identified as ‘shortfall nutrients’ by the DGA [1]. In addition, the current intakes of fiber and potassium are low to the extent that they may pose a public health concern and therefore are termed ‘nutrients of public health concern’ by the DGA [1]. Less than 5% of the population is currently consuming more than the adequate intake of dietary fiber or potassium [1]. The DGA indicates that the low intakes of dietary fiber and potassium are due to low intakes of fruit and vegetables, and recommends eating more fruit and vegetables along with whole grain and dairy to increase intakes of fiber and potassium as well as other nutrients of public health concern [1]. Raisin consumers had lower intakes of added sugars, saturated fat, and sodium compared to non-consumers. These are called ‘nutrients to limit’, and eating patterns that are low in these nutrients are recommended by the DGA [1].

The raisin consumers also had higher intakes of fruit, whole fruit, vegetables, and whole grain. Higher intakes of these nutrient-dense food groups are indicative of overall healthier diets. The HEI is a validated measure of diet quality commonly used to evaluate diets including subpopulations [27] and food environments [28], to assess changes in diet quality over time [29] and the efficacy of dietary interventions, and to validate other nutrition research tools and indices [30]. It has also been used in research to understand relationships between nutrients, foods, and dietary patterns and health-related outcomes [31–34]. It also indicates compliance or adherence of the diets to the dietary recommendations and has 12 components (nine for adequacy and three for moderation), each of which relates to the key recommendations of the DGA 2010 [35]. Indeed, the HEI-2010 total scores as well as the scores of most subcomponents (nine subcomponents out of 12) of raisin consumers were significantly higher than those of non-consumers. Higher diet quality of consumers of grape products (fruit, juice, and raisins) compared to non-consumers was also reported in the earlier cross-sectional study using NHANES 2003–2008 data [16]. An earlier analysis of NHANES 1994–2004 also reported higher diet quality among consumers of dried fruits [4]. However, that analysis included raisins as well as non-raisin dried fruits [4].

In the current study, raisin consumers had lower weight, BMI, and waist circumference than non-consumers, and raisin consumption was significantly associated with lower odds of being overweight or obese and having elevated waist circumference. Intake of dried fruits was associated with lower weight, BMI, and waist circumference in an earlier cross-sectional study [4]. This finding may have important health and economic implications, as more than one-third of US adults are obese [36] and obesity is associated with several health risks [37] and annual medical costs of $147 billion [38]. Mechanisms related to the potential benefits of raisin intake for weight management have not been critically explored. A few intervention studies did not find any effect of raisins on body weight in people with type 2 diabetes [9] or in overweight or obese subjects with elevated glucose [10]. Small clinical studies suggest that raisins may increase satiety and decrease appetite [14,15]. However, as energy intake was significantly higher (energy balance was not measured), the current findings of lower body weight, BMI, and waist circumference, and lowered risk for obesity and related parameters, for the raisin consumers need to be further investigated, especially in a healthy population. It is possible that there is differential underreporting of energy intake by raisin consumers and non-consumers, and also that raisin consumption may just be a marker for those who lead a healthier lifestyle (e.g., less sedentary behavior).

Raisin intake was not associated with many physiological measurements, including BP, blood lipids, or blood sugar/insulin in the present analysis. Human intervention studies have reported mixed results on the effects of raisins on BP [9–11]. Raisin intake did not affect the lipid profile of fasting glucose or insulin in clinical studies [9–11]. However, raisins have consistently been demonstrated to lower postprandial glycemia and/or insulinemia in acute human feeding studies with healthy and diabetic subjects [10–13]. Raisins are high in fructose, which has a low glycemic index [13]. Although there were no differences in levels of CRP between consumers and non-consumers, the raisin consumers had a significantly reduced risk of having elevated CRP (OR = 0.05, *p* < 0.0001) in the present study. CRP is a sensitive marker of inflammation, and elevated levels have been associated with the risk of coronary artery disease and metabolic syndrome [39,40]. Raisin consumers were also at a 54% lower risk of metabolic syndrome.

Overall, it is not surprising that the intake of added sugars was lower in raisin consumers as it is likely that raisins replaced other sources of sugars and added sugars in the diet. The higher intakes of total vegetables and whole grains, along with the lower intakes of saturated fats and sodium (and the lower smoking rates and less sedentary behavior) support the concept that the raisin consumption is a marker for a healthier lifestyle.

### Strengths and limitations

A limitation of this study is that cross-sectional studies cannot be used to determine cause and effect. In addition, 24 h dietary recalls rely on participants’ memory to self-report dietary intakes and are subject to misreporting. Also, the data used in this study were based on a single 24 h dietary recall and may not represent usual intake; that said, linking a single dietary recall to physiological measurements should bias results to the null. Strengths of this study include the use of large nationally representative sample achieved through combining several sets of NHANES data releases, and the use of numerous covariates to adjust data to remove potential confounding. The researchers, however, acknowledge that residual confounding may still exist that could explain some of the results reported.

## Conclusion

The results of this study suggest that raisin consumption is associated with better nutrient intake, diet quality, and weight parameters, and with lowered risk of obesity and metabolic syndrome in US adults, and may be a marker for those who lead a healthier lifestyle.
